# Targeted imaging of orthotopic prostate cancer by using clinical transformable photoacoustic molecular probe

**DOI:** 10.1186/s12885-020-06801-9

**Published:** 2020-05-14

**Authors:** Chen Qiu, Yuanyuan Bai, Tinghui Yin, Xiaoyan Miao, Rongkang Gao, Huichao Zhou, Jie Ren, Liang Song, Chengbo Liu, Hairong Zheng, Rongqin Zheng

**Affiliations:** 1 Department of Medical Ultrasonic, The Third Affiliated Hospital of Sun Yat-sen University, Guangzhou, 510630 China; 2 Research Laboratory for Biomedical Optics and Molecular Imaging, Shenzhen Institutes of Advanced Technology, Chinese Academy of Sciences, Shenzhen, 518055 China; 3 Paul C. Lauterbur Research Center for Biomedical Imaging, Institute of Biomedical and Health Engineering, Shenzhen Institutes of Advanced Technology, Chinese Academy of Sciences, Shenzhen, China

## Abstract

**Background:**

To obtain high-yield histological samples by targeted prostate cancer (PCa) biopsy is the current trend compared with transrectal ultrasound (TRUS)-guided systematic histological biopsy, which is regarded as the gold standard for prostate cancer (PCa) diagnosis. In this paper, we present a targeted PCa imaging strategy using a real-time molecular photoacoustic imaging system integrated with a handheld US probe (PAI/US) and synthesized an integrin *α*_*v*_*β*_*3*_ targeted probe based on ICG (cRGD–ICG).

**Methods:**

To prepare cRGD–ICG, ICG-NHS was linked to cRGD through carboxyl-co-reaction. In vitro PA imaging ability of cRGD–ICG was tested. Orthotopic PCa-bearing rats were used as animal models. After injected with either cRGD–ICG or non-targeted probe, rats were implemented with PA imaging to confirm the specific accumulation of cRGD–ICG at tumor region. Moreover, pathological frozen slices were made to observe distribution of the probe in prostate tissue ex vivo.

**Results:**

A small molecular PAI probe was synthesized and exhibited excellent targeted imaging ability in vitro. In vivo photoacoustic imaging was carried out after intravenous injection of cRGD-ICG in orthotopic PCa-bearing rats under the facilitation of the PAI/US system. Maximum molecular photoacoustic signals were observed in the tumor area in vivo after the probe injection, which showed 3.8-fold higher signal enhancement than that in the control group (*P* < 0.05). Significantly higher cRGD-ICG accumulation was observed under confocal microscopy in the tumor region than in normal prostate tissue.

**Conclusions:**

All our results showed that the comprehensive strategy provided a high-yield and reliable method for PCa diagnosis and targeted prostate biopsy, with great clinical translation potential.

## Background

Prostate cancer (PCa) is the most common malignancy and is the third highest cause of death among the tumor-bearing male population in the united states [1]. Universally, systematic histological biopsy guided by transrectal ultrasound (TRUS) has been regarded as the gold standard procedure for diagnosing PCa [2]. However, since the sensitivity and specificity of the US method to identify malignancies are relatively low, US is mainly used to provide anatomical reference within the prostate while performing a systemic biopsy. In addition, systemic biopsy is used dependent and may result in inconsistent sampling [3], which often leads to missing clinically significant prostate cancer. Thus, to overcome the limitations of systematic biopsy, targeted biopsy techniques are being explored, of which multiparametric magnetic resonance imaging (mpMRI) is the most commonly used [4]. In the mpMRI technique, suspicious regions identified in MRI are targeted for biopsy under US guidance using fusion imaging. However, this technique is still too expensive to afford, along with a 5–15% false-negative cancer detection rate [5]. Therefore, this approach is limited by the detectability of PCa-targeted biopsy.

US molecular imaging is also a complementary tool to enhance the accuracy of PCa diagnosis while using US. A first-in-human US molecular imaging trial with BR55 as a vascular endothelial growth factor receptor 2-targeted contrast agent has shown promise for the specific detection of human PCa [6]. However, since the missed lesion rate is as high as 32%, this approach still cannot be used clinically, and its feasibility for diagnosing PCa needs to be further explored [7]. Thus, it remains a challenge to search for an imaging modality that can reliably distinguish benign from malignant tissue of the prostate for improved PCa detection [8].

Photoacoustic imaging (PAI) is an emerging clinical modality that combines a high sensitivity inherent to optical properties, as deep as 5 cm, and a high spatial resolution inherent to US imaging [7]. The photoacoustic imaging system can be easily integrated into the clinical US imaging platform without degrading the inherent US imaging capability. Hence, the PAI modality has provided unprecedented opportunities for PCa diagnosis. Both preclinical and clinical studies have already demonstrated the ability of PAI to localize tumors [8–10]. Furthermore, as in other mainstream imaging modalities such as US, CT or MRI, tumor cells can be prelabeled with photoacoustic molecular probes for contrast-enhanced imaging. The combination of PAI and molecular probe targeting and labeling, also called photoacoustic molecular imaging, has shown great potential to identify malignances, including PCa. A few studies with molecular PAI have already been performed to identify PCa, especially in orthotopic PCa models, which mimic the heterogeneous tumor microenvironment, foretelling the feasibility of clinical translation [11, 12].

For molecular PAI, exogenous contrast agents including nanoparticles and dyes are commonly used to provide sufficient contrast in the signal. Specifically, the small molecular dye indocyanine green (ICG) has great translational potential for clinical PAI because of its high biosafety level. ICG is a near-infrared fluorescent dye already approved by the US Food and Drug Administration (FDA) for clinical imaging applications [13]. Consequently, ICG is currently used with PAI clinically, such as identifying sentinel lymph nodes, which is critical for cancer staging [10]. However, free ICG has several drawbacks for in vivo imaging, including a tendency to aggregate, instability in aqueous solution, rapid clearance from the bloodstream, and lack of targeting abilities [14, 15]. Thus, ICG combined with stable and targeted molecular probes is needed for PCa since free ICG alone cannot be used for targeting PCa with PAI.

The cell adhesion molecule integrin *α*_*v*_*β*_*3*_ which interacts with RGD motifs of the extracellular matrix is known to play a critical role in tumor growth, angiogenesis and metastasis in several tumor types including PCa by modulating cell adhesion, proliferation and migration [16, 17]. Molecular probes linking RGD, namely, the arginine-glycine-aspartic acid sequence, with the *α*_*v*_*β*_*3*_ integrin receptor would enable noninvasive monitoring of tumor angiogenesis and metastasis using PAI [18]. This ICG-based molecular probe would be an ideal candidate for PCa management with PAI.

In the current study, we present a photoacoustic molecular imaging-based technique to identify malignancies by enhancing both functional and molecular information of PCa using a synthesized molecular probe to increase the detectability of prostate cancer for better US-based targeted prostate biopsy, overcoming the limitations of previous targeted methods.

## Methods

### Synthesis and characterization of cRGDyk-conjugated ICG

#### cRGD-ICG synthesis

**Fig. 1:**
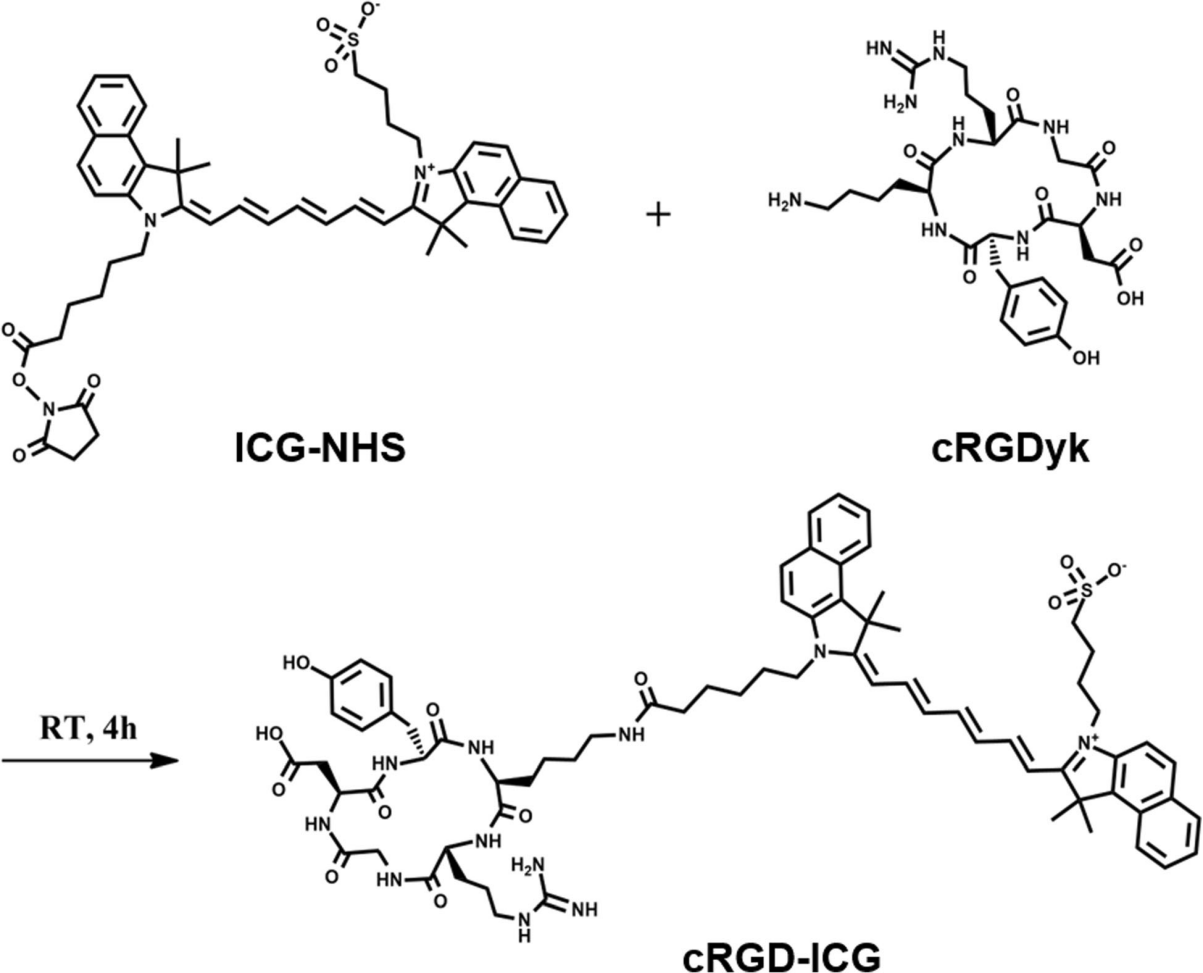
Schematic structures of ICG conjugated c(RGDyk) probes

Monomeric cyclic RGD peptide c(RGDyK) was used (Shangon, China) for the synthesis of the probe. Appropriate c(RGDyK) was mixed with ICG-NHS ester (Kaixin, China) in a molar ratio of 1:3 by dissolving in dimethyl sulfoxide (DMSO). The reaction mixture was incubated for 4 h in the dark at room temperature. Subsequently, the mixture was diluted to 3 ml by adding water. The solution was then transferred to a dialysis device for 48 h at 4 °C to remove free ICG. The concentrated c(RGDyK)-ICG solution was subsequently lyophilized to remove organic solvents. Finally, the powder of c(RGDyK)-ICG was dissolved in water and stored at − 20 °C in the dark until use. Brief procedures describing the cRGD-ICG synthesis are shown in Fig. 1. The synthesized cRAD-ICG was prepared as a negative control by linking ICG to the nonfunctional cRAD peptide.

#### Characterization of cRGD-ICG

Ultraviolet-visible (UV-Vis) spectra and fluorescence spectra of the probes were obtained by using a UV-Vis spectrophotometer (Evolution 220, Thermo Scientific, USA) and a fluorescence spectrophotometer (Lumina, Thermo Scientific, USA), respectively. In vitro photoacoustic imaging of the probes was performed using a handheld US linear array-based photoacoustic imaging system (Shenzhen Institutes of Advanced Technology, Chinese Academy of Sciences, Shenzhen). A series of different concentrations of probes (1.25–20 μg/ml) were placed in 96-well plates by adding 200 μl of solution to each well. Further quantitative photoacoustic signals of the probe were obtained using MATLAB software.

### Cell culture

Human umbilical vein endothelial cells (HUVECs) expressing *α*_*v*_*β*_*3*_ integrin and human embryonic kidney cells (293 T) barely expressing integrin *α*_*v*_*β*_*3*_ were permitted and purchased from the Cell Bank of Shanghai Institutes for Biological Sciences, Chinese Academy of Sciences (Shanghai, China). Both cell lines were grown in Dulbecco's Modified Eagle's Medium (DMEM) containing 10% fetal bovine serum (FBS) at 37 °C in a humidified atmosphere with 5% CO_2_. When the cells reached 70% confluence, they were trypsinized and subcultured. All cell culture reagents were obtained from Invitrogen Corporation (Carlsbad, CA, USA). Cells were seeded in 6-well plates at a density of 1 × 10^5^ cells per well and were incubated overnight in complete cell growth medium. The 0.5 ml medium containing either cRGD-ICG or cRAD-ICG was incubated with the cells for 1 h at 37 °C. The medium was then changed back to complete cell growth medium for normal cell culture before conducting binding affinity experiments.

### Cell-specific uptake of cRGD-ICG assay

To examine the intracellular uptake of cRGD-ICG or cRAD-ICG, both HUVECs and 293 T cells were incubated with either cRGD-ICG or cRAD-ICG (20 μg/ml) for 1 h at 37 °C. After incubation, unbound dyes were removed with a gentle PBS wash, and the medium was replaced before imaging. With the obtained final solution, flow cytometry (Accuri C6, BD, USA) was performed to determine quantitative binding through the ICG signal.

Cells also underwent a similar procedure to test the cellular targeting ability of PAI. Once cell solutions were collected, they were subcutaneously injected into the backside of a normal murine model and were observed with a photoacoustic system (Shenzhen Institutes of Advanced Technology, Chinese Academy of Sciences). Further quantitative data were obtained offline using MATLAB software.

### Murine model of orthotopic prostate cancer

All animal experiments were approved by the Sun Yat-sen University Animal Care Committee. All experimental rats were purchased from Charles River (Beijing, China). A male nude rat (Sprague-Dawley rat) bearing an orthotopic xenografted PCa model was established. The operation procedures were as follows: 12 mature rats weighing 250–300 g were used for the study. All rats were kept in a temperature- and humidity-controlled room and were randomly divided into 2 groups: the cRGD-ICG and cRAD-ICG groups (*n* = 6 in each group). The rats were fasted 18 h before the operation but were fed chow and water otherwise. The experiment began after the rats were appropriately anesthetized by injecting pentobarbital sodium (30 mg/kg, i.p.). Then, longitudinal incisions were made in the lower abdomen to expose the prostate. A 50 μl human prostatic carcinoma cell line (PC-3) suspension at 5 × 10^6^/ml was then injected into the gland. The incision in the abdomen was closed, and successful orthotopic PCa modeling was confirmed 3 days after the operation by pathology (further details are provided in the supplemental materials and methods).

### In vivo PAI molecular imaging

Rats in each group were injected with 200 μl cRGD-ICG or cRAD-ICG (50 μg/ml) solution via tail vein. Imaging was performed at a series of time points after injection: 0 h, 5 min, 2 h, 4 h, 8 h, and 12 h. We used the PAI/US dual-modality imaging system with the following details: 7 MHz linear array transducer (Blatek, USA) with 128-element (pitch 3 mm), lateral and axial resolution of 336 and 235 μm, respectively, 15 mJ/cm^2^ laser fluence, 795 nm wavelength, 6 ns laser pulse width, and 20 Hz pulse repetition frequency. Further details of the imaging system used can be found in our previous publications [19, 20]. The reconstruction algorithm was based on back-projection algorithm. The transducer surface was maintained at approximately 9 mm from the surface of the skin and was coupled with clear and colorless US gel for uniform light illumination to the gland and for consistent imaging between different time points. B-mode US images were used as a reference to maintain consistent imaging planes between different imaging time points and for general anatomy information.

### Analysis of in vivo PAI imaging data

We quantified the integrin *α*_*v*_*β*_*3*_-targeted PAI signal using a MATLAB-based algorithm. The engineer was blinded to group assignment and outcome. The coregistered B-mode US images were used as guidance to select the region of interest (ROI) in the prostate gland comprising both normal and cancerous tissues. Within the identified ROI, PAI signals identifying PCa tissues were analyzed. The quantitative PAI signal was presented as the ratio of pixels containing molecular signal postinjection compared to the signal preinjection. In addition, absolute quantitative PAI signals at each point of imaging depth were calculated.

### Histological confirmation of ICG localization in murine prostate cancer

Twelve hours postinjection, animals were euthanized by injecting excessive pentobarbital sodium 100 ~ 150 mg/kg. Afterwards, the whole prostate including the tumor was resected and frozen at the optimal cutting temperature (O.C.T.) for pathology (see details in the supplemental materials and methods). A confocal laser scanning microscope imager (Leica, Germany) was used to acquire fluorescent images of tissue sections. The ICG component in the molecular probe can be seen in photoacoustic images in vivo as well as in fluorescence images of tissue sections. The pathology of suspicious accumulation of the cRGD-ICG region, identified as PCa in real-time PAI imaging, was confirmed by the pathology results. Tissue sections (10 μm thickness) of the two groups were rinsed in PBS for 5 min to remove the O.C.T. compound and then fixed for 5 min in 4% PFA. Slides were then imaged using PA at 405 nm for the nuclei and 633 nm for ICG.

### Statistical analysis

All experiments were repeated 3 times, and the data were analyzed using Student’s t-tests and one-way ANOVA (SPSS software, version 13.0, SPSS Inc.). Unless otherwise specified, data were expressed as the means ± standard deviation. A two-sided *P*-value of less than 0.05 was considered indicative of a statistically significant difference in values.

## Results

### Synthesis and characterization of cRGD-ICG

As described before, brief procedures of c(RGDyK)- ICG synthesis by amide linkage are shown in Fig. 1. NHS ester reacted with the *ε*-amino group of the lysine residue of the RGD peptides.

**Fig. 2:**
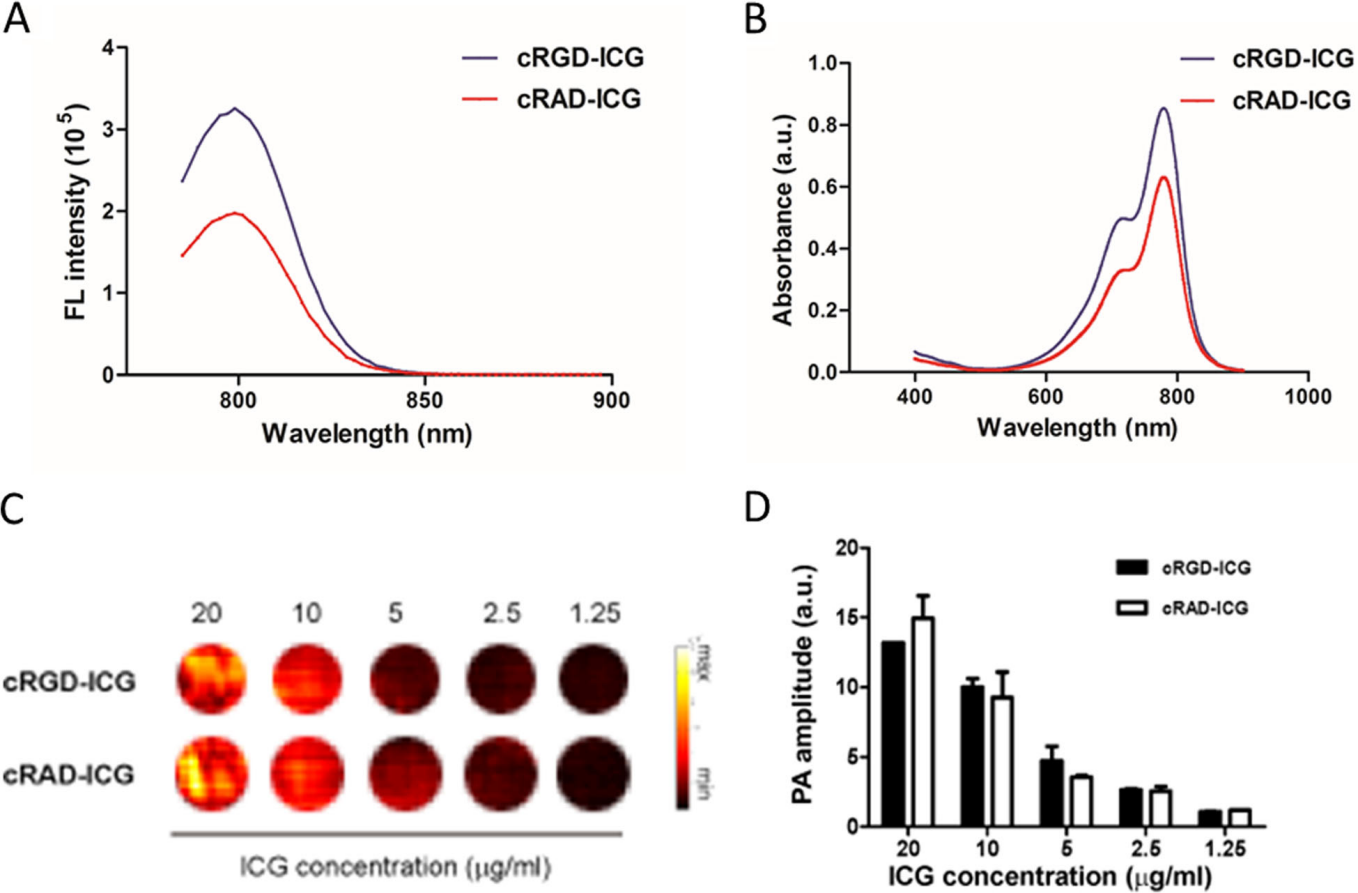
haracterization of cRGD-ICG. **a** Emission spectra of both cRGD-ICG and cRAD-ICG. **b** Absorbance spectra of both cRGD-ICG and cRAD-ICG. **c** In vitro photoacoustic images of cRGD-ICG and cRAD-ICG. **d** In vitro photoacoustic amplitude signal of a series of concentrations cRGD-ICG and cRAD-ICG

In buffer, as shown in Fig. 2a and b, the fluorescence emission and absorption characteristics of the targeted cRGD-based dyes and nontargeted dyes were similar. The maximum emission of light for cRGD-ICG is at 799 nm (Fig. 2a), and the specific absorption wavelength of cRGD-ICG is at 795 nm (Fig. 2b), where PAI imaging will be optimum. At 795 nm, the in vitro PAI properties of the molecular probe were tested at different concentrations (1.25–20 μg/ml), and the results are shown in Fig. 2c and d. cRGD-ICG displayed a stable linear PAI signal increase as the concentration was increased linearly from 1.25 to 20 μg/ml.

### Cell-specific uptake in the cRGD-ICG assay

**Fig. 3:**
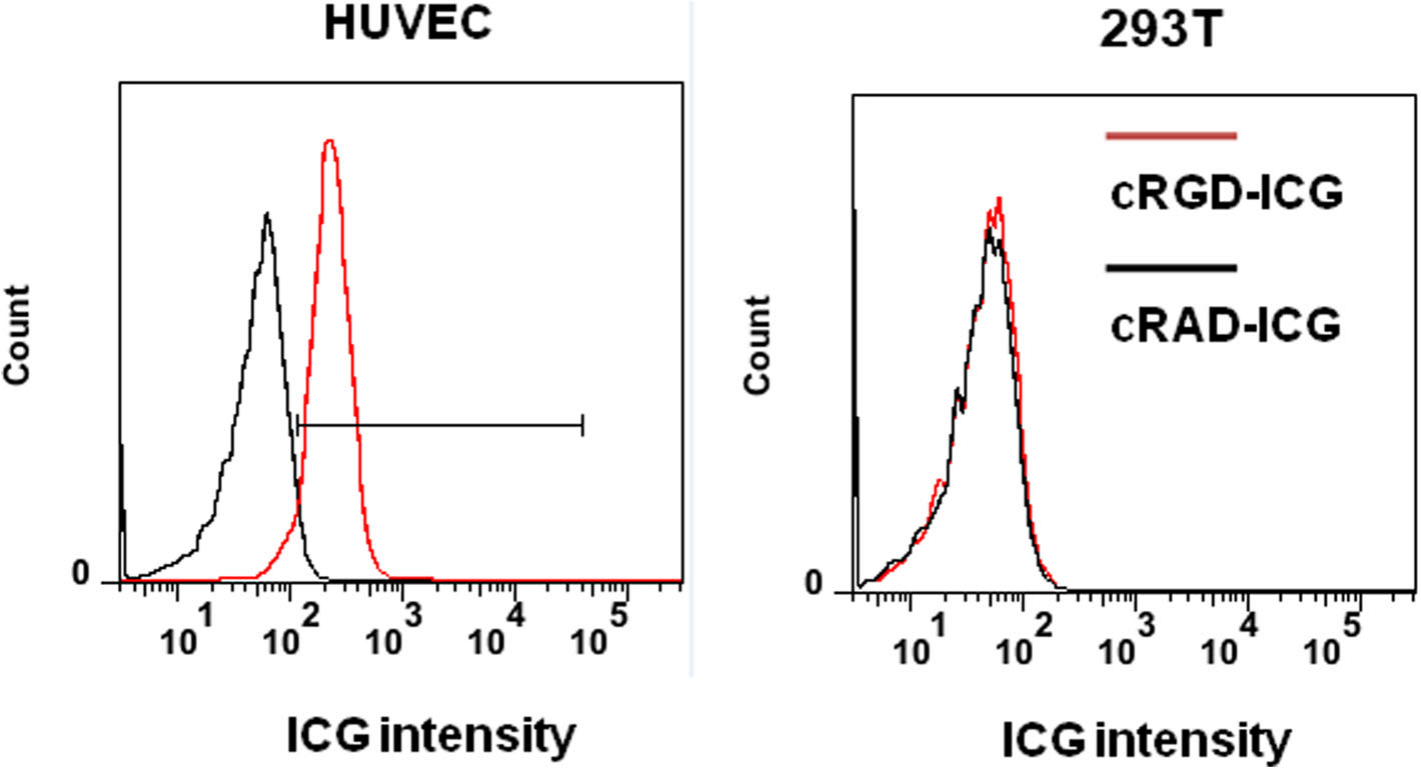
Cell specific uptake of cRGD-ICG by flow cytometry. Red curves: HUVECS or 293 T incubated with cRGD-ICG. Black curves: HUVECS or 293 T incubated with cRAD-ICG

To examine the binding affinity of cRGD-based probes towards the integrin *α*_*v*_*β*_*3*_ receptor in vitro, we tested both the HUVEC cell line and 293 T cell line (as a control). After incubating 50% of HUVECs with a targeted agent (cRGD-ICG) and another 50% with a nontargeted agent (cRAD-ICG), cells were collected for flow cytometry. As shown in Fig. 3, HUVECs with abundant integrin *α*_*v*_*β*_*3*_ receptors exhibited much stronger ICG signals when incubated with cRGD-ICG than when incubated with cRAD-ICG. The quantitative analysis showed that the binding efficacy of cRGD-ICG towards HUVECs was 93.8 ± 5.7% (*P* < 0.05), which confirmed the excellent targeting ability of cRGD-ICG, laying the foundation for in vivo molecular imaging. In the control cell groups, 293 T cells with a very low expression of integrin *α*_*v*_*β*_*3*_ receptors barely displayed ICG signals when incubated with cRGD-ICG or cRAD-ICG. In addition, the difference in cellular uptake for 293 T cells was not statistically significant between cRGD-ICG and cRAD-ICG (*P* > 0.05).

**Fig. 4:**
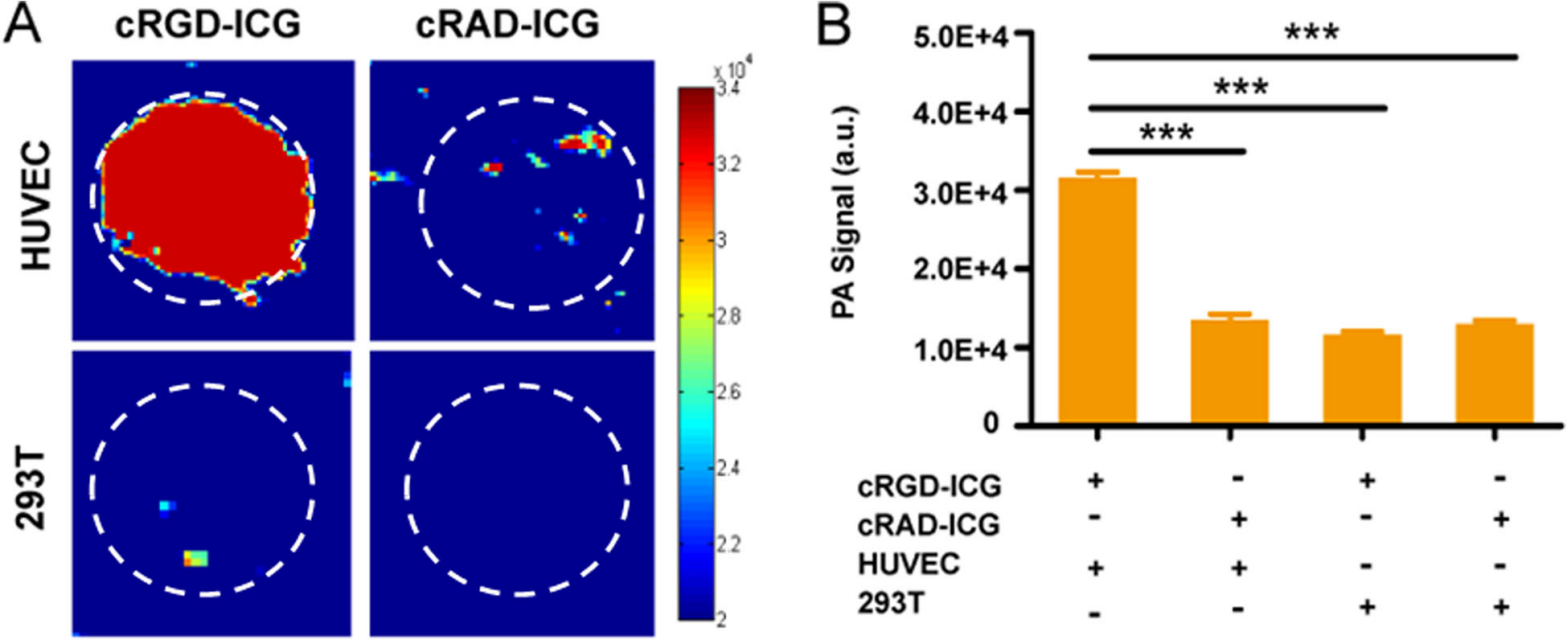
Photoacoustic signals of cell inoculation. **a** Cellular photoacoustic images. **b** Cellular photoacoustic amplitude signal analysis

We then conducted live-cell PAI using the same cell lines to verify the cellular targeting ability. Cells incubated with probes were injected subcutaneously into the dorsal side of a murine model to facilitate PAI. As shown in Fig. 4, HUVECs belonging to the cRGD-ICG group showed a significant PAI signal, while those belonging to the cRAD-ICG group showed a very low PAI signal (Fig. 4a). The 293 T cells, independent of the targeted or nontargeted probe, showed very low molecular signals. The quantitative analysis (Fig. 4b) showed that the HUVECs belonging to the cRGD-ICG group achieved almost 2.4-fold enhanced PAI signal intensity compared to that of the control groups (*P* < 0.05). This result demonstrates the targeting ability of cRGD-ICG at the cellular level for enhanced PAI imaging.

### Dynamic molecular PAI of cRGD-ICG in an orthotopic prostate cancer in vivo

**Fig. 5:**
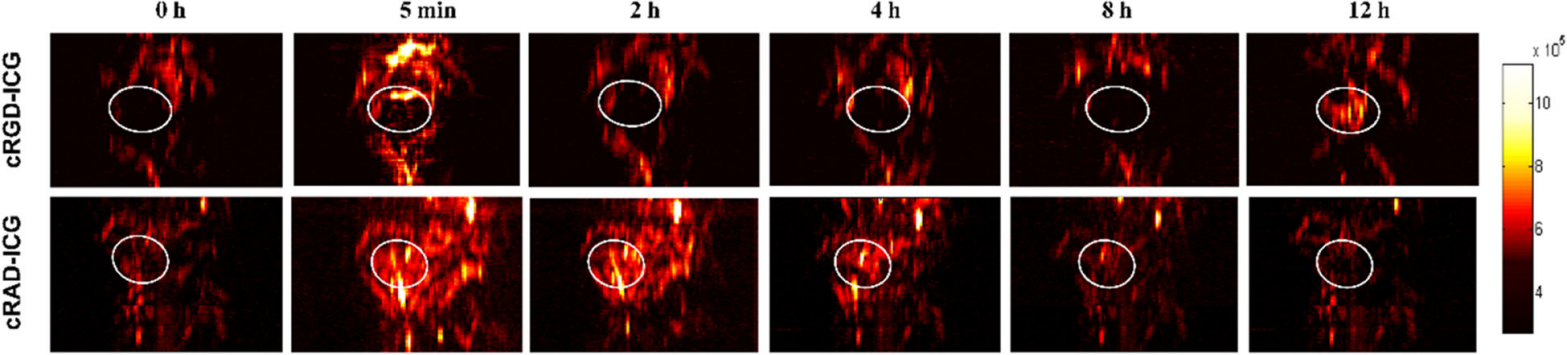
PAI of rats are bearing orthotopic PCa after injected with cRGD-ICG or cRAD-ICG for a period of time. White circle: suspicious tumor area in the prostate

The successful murine orthotopic prostate tumor model is shown in Fig. S1, and the tumor sections collected to confirm the expression of integrin *α*_*v*_*β*_*3*_ are shown in Fig. S2. To assess the feasibility of using the *α*_*v*_*β*_*3*_ molecular probe for murine orthotopic PCa targeting, cRGD-ICG was injected into 6 rats via the tail vein. cRAD-ICG was injected into the remaining 6 rats and was used as a negative control probe. PAI was performed at a series of time points before and after probe injection to better understand the physiological behavior of cRGD-ICG. PAI imaging of the abdominal region of the murine models was performed with rats laying on their dorsal size and the transducer placed anteriorly. The maximum amplitude projection images of one of the rat’s abdomens are shown in Fig. 5. In the top row of Fig. 5, it can be seen that cRGD-ICG accumulated temporally (for 12 h after probe injection) and spatially at a suspicious tumor area in the prostate. The malignancy of the tumor area was further confirmed by pathology (Fig. 7). Other rats within the cRGD-ICG group showed a similar tendency, as shown in the left column of Fig. S3. For the control rats injected with cRAD-ICG, no obvious probe accumulation occurred in the prostate tumor area, as shown in the bottom row of Fig. 5 and the right column of Fig. S3.

### Molecular PAI/US of orthotopic prostate cancer

**Fig. 6:**
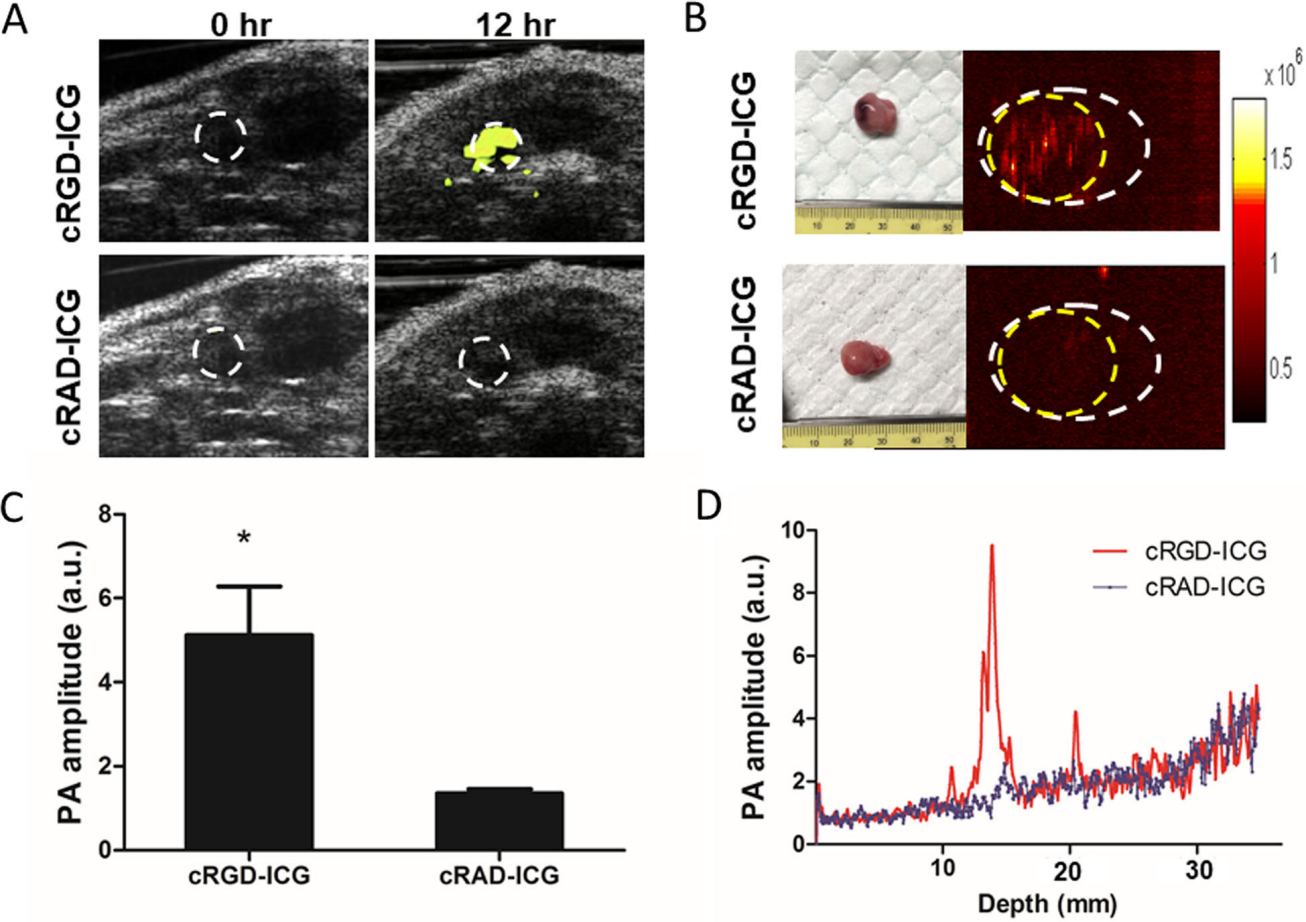
Integrin *α*_*v*_*β*_*3*_ targeted PAI of orthotopic PCa. **a** In vivo integrin *α*_*v*_*β*_*3*_ PAI images merged with US images. **b** Ex vivo PAI images of prostate harvested at 12 h. Yellow circle: tumor area. White circle: the whole prostate including tumor and normal tissue. **c** PAI signal enhancement after injection with dye. **d** PAI signal varied from depths at 12 h

Molecular PAI/US imaging was performed at the optimum imaging time (12 h after probe injection) to identify PCa. As shown in Fig. 6a, the PAI images show integrin *α*_*v*_*β*_*3*_ molecular signals merged with B-mode US images, providing valuable anatomical information around the tumor. The rats injected with cRGD-ICG showed significant molecular PAI signal enhancement at the orthotopic tumor area 12 h postinjection, with US showing the surrounding tumor structures (Fig. 6a). In the rats injected with cRAD-ICG, only sparse molecular PAI signal distribution was observed in the same region (Fig. 6a). The prostate area with PCa showed an average 5.1-fold increase in the molecular PAI signal but only a 1.3-fold increase in the control group after probe injection (*P* < 0.05) compared to the baseline preinjection signal (Fig. 6c). The quantitative PAI signals at different depths, from the skin to deep tissues, were also measured to check the location of the maximum PAI signal. As expected, PAI signals of the cRGD-ICG group peaked at a depth of 10 mm, which was the orthotopic PCa area (Fig. 6d).

The prostate tissues, including the tumor area, were excised 12 h postinjection for ex vivo PAI (Fig. 6b). In the cRGD-ICG group, the *α*_*v*_*β*_*3*_ molecular PAI signal was confirmed to accumulate mostly in the tumor region with no significant signal accumulation in the normal prostate region (Fig. 6b). In the cRAD-ICG group, whole prostate tissues, including the tumor region, showed a low PAI signal.

### Histological confirmation of ICG localization in murine prostate cancer

**Fig. 7:**
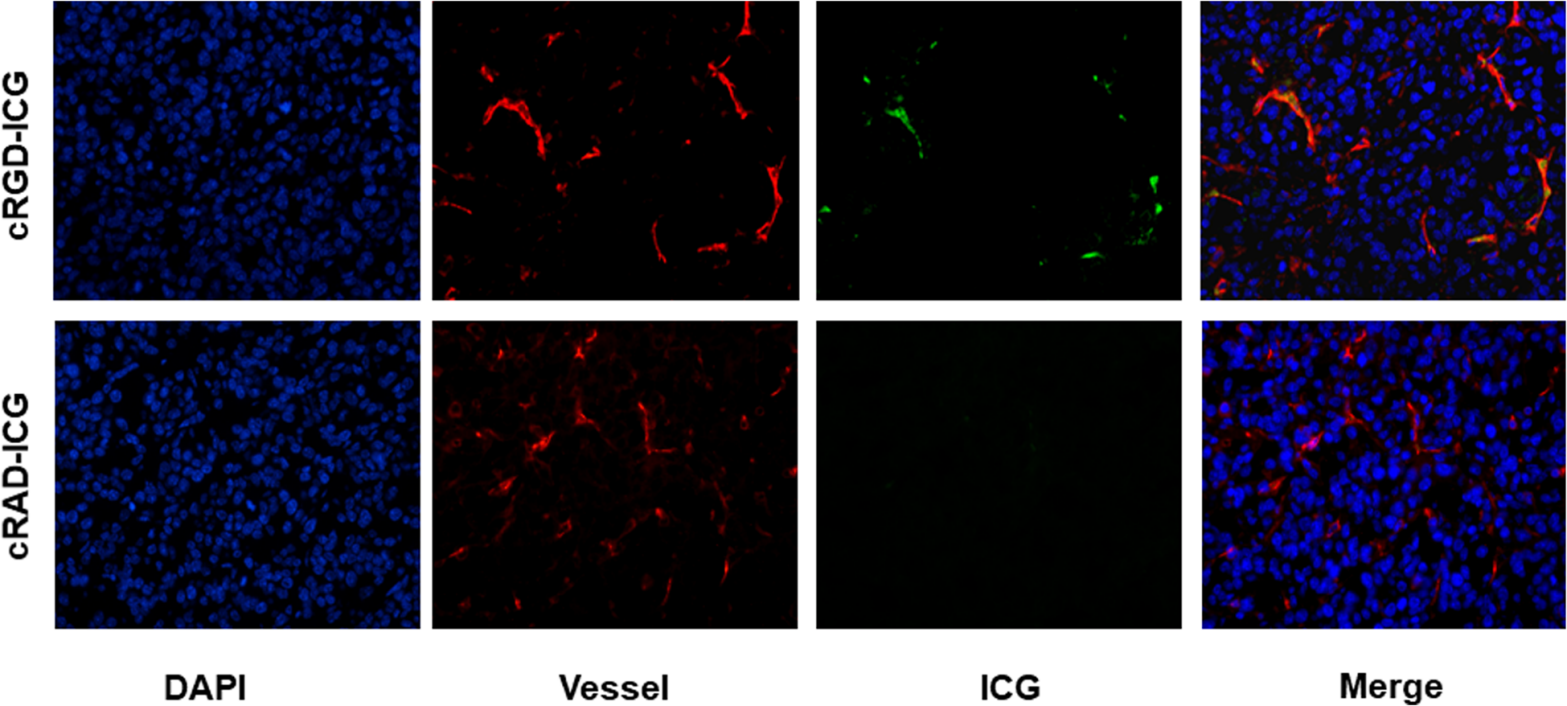
Confocal microscope imaging of ICG in tumor tissue. Blue fluorescence: nuclei stained with Hoechst 33342; Red fluorescent: vessel stained with FITC; Green fluorescent: ICG. (Magnitude: 400 ×)

To confirm the accumulation of the probes within tumor tissues, the prostate cancer regions were excised 12 h after dye injection for frozen slices. cRGD-ICG and cRAD-ICG were visualized in frozen slices using a confocal microscope to acquire fluorescence signals from ICG. The neovascularization within the tumors was labeled with red fluorescence. As expected, abundant ICG signals (green in the image) distributed along tumor neovascularization in the cRGD-ICG group were detected (Fig. 7). This result using microscopy confirms the binding affinity of cRGD-ICG to neovascularization of PCa for targeting. For the cRAD-ICG control group in vivo, ICG was barely found adhering to the vessels due to the poor targeting ability of the probe.

## Discussion

Systematic biopsy for prostate cancer detection, which is currently the gold standard to evaluate PCa, suffers from several limitations including low accuracy, uneven sampling, which can lead to undersampling of clinically significant prostate cancer under diagnosis, and complications. As presented in the introduction, mpMRI and molecular US imaging are investigated for targeted biopsy to improve the efficiency of the core biopsy but have limitations [5, 6]. Determining how to improve the targeting ability during image-guided biopsy remains a challenging issue. On the other hand, even though conventional US only aids in providing anatomical information of the prostate, the real-time convenience and reproducibility still make it a valuable guidance tool. In addition, the general anatomical information provided by US between the prostate and surrounding tissues, such as the bladder and urethra, helps in reducing procedure injury. Therefore, the optimal way to address the targeted prostate biopsy is to find a targeting method that is complementary to US imaging while increasing the success of the prostate biopsy.

The PAI system integrated with the US system provides such an opportunity to improve the efficacy of prostate biopsy. Photoacoustic molecular imaging compliments US imaging since it enables monitoring of the disease process with additional molecular and functional information in addition to providing anatomical structural information. The PAI is more accurate in distinguishing malignancies, as reported in several studies [8–10]. The PAI system can be easily integrated into the clinical US imaging platform without degrading the inherent US performance which shows great potential for clinical translation [21].The fastest-growing molecular PAI overcomes the sensitivity limitation inherited from US and has an excellent resolution at a satisfactory imaging depth for targeted PCa application.

In this study, we presented a molecular PAI targeting of PCa using synthesized ICG, an FDA-approved small molecular dye. Targeting substances such as peptides and proteins linked to the dye have been evaluated in the past to achieve targeting ability. In fact, proteins (for example, monoclonal antibodies) could achieve higher targeting affinity and specificity than the peptide used in this study but have poorer tissue penetration and clearance due to their larger size, hindering their wider application. Thus, the molecular probe synthesized in this study showed greater clinical translation potential.

A few other studies have focused on molecular PAI to detect PCa in vivo. Ni et al. monitored PCa growth in an orthotopic mouse model through 3D US-assisted PAI functional imaging by evaluating the angiogenesis feature of the tumor instead of molecular information [22]. Two similar studies [11, 12] evaluating the utility of targeted PAI for PCa also based on small molecule dye. Both of their results showed significant PAI signal enhancement after targeted probe injection. As also depending on PC-3 prostate tumors, study of Levi et al. presented nearly 2-fold PAI signal increase while about 5-fold in our study. Besides, both of the studies used subcutaneous xenograft tumor models of nude mice which couldn’t highlight the depth advantage of PAI. In the present study, an orthotopic xenografted tumor model in rats was used instead of the subcutaneous tumor model to mimic the heterogeneous tumor microenvironment and to take full advantage of the imaging depth of PAI. Our study setting aligns more with the clinical setting, thus reflecting the significance for clinical translation.

In this study, we also demonstrated the depth of penetration advantage of PAI in the rat orthotopic PCa model. Even with a certain distance between the skin and the tumor area, excellent molecular PAI images were successfully obtained for the whole prostate gland. The molecular PAI signal in the cRGD-ICG group peaked at a depth of 10 mm, where the orthotopic tumor was located, demonstrating the targeting ability of the probe. In fact, the depth of penetration of PAI is more than 5 cm, which meets basic clinical needs.

In the present study, PAI and US imaging were merged to copresent orthotopic PCa and the surrounding anatomical area, including the prostate and bladder. This anatomical information is vital not only for avoiding injury to normal tissues during biopsy procedures but also for identifying PCa areas in PAI. The merged PAI and US images identified PCa for targeted biopsy, aided by the critically important targeting ability of the molecular probe towards tumor angiogenesis. We quantitatively analyzed the molecular imaging results of PCa in this study. The rats injected with the targeted probe showed a 3.8-fold higher (*P* < 0.05) PA signal in the tumor region than that of rats injected with the nontargeted probe, which validates the significant targeting ability of our molecular PAI for PCa. The merged imaging method enabled visualizing and quantifying tumor malignancy at a molecular level and increased the sensitivity of diagnosing the tumor, which will potentially increase the accuracy of targeted biopsy and even local treatment for PCa clinically.

Notably, there are some limitations of our study. First, the targeting probe that we used in this study to visualize PCa is a general molecule that is overexpressed in a wide range of diseases. While we were successful in clearly identifying PCa, a probe specifically targeting PCa would be more than welcome. We will investigate such a probe in the near feature. Second, the animal model and molecular probe we used in the current study mainly addressed diagnosis of advanced PCa. The diagnosis value of primary PCa remains is therefore uncertain. Molecular PAI targeting primary PCa should be further explored. Third, the development of PA-based multimodal contrast agents that can provide complementary information from various different imaging modalities is still warranted. In the absence of such a contrast agent, our ICG-based contrast agent is a valuable targeting agent for PCa diagnosis in multimodal PAI. Lastly, to obtain targeted prostate biopsy by dual PA/US imaging, a transrectal system will be closer to clinical translation. In the near future, we will develop PAI configuration combines a transrectal US transducer with the coupling of effective laser excitation on a canine model.

## Conclusions

In this study, we successfully synthesized an integrin *α*_*v*_*β*_*3*_-targeting PAI probe by combining the favorable photoacoustic properties of ICG and specific *α*_*v*_*β*_*3*_-binding capabilities (cRGD-ICG). Employed with cRGD-ICG, molecular PAI showed excellent diagnostic ability for murine orthotopic PCa models by PAI/US merged imaging. The strategy that we developed in this work showed great potential for guiding targeted prostate biopsy towards highly suspicious regions, thus increasing the biopsy rate during systematic biopsy. Because the dye is already FDA approved and the PAI depth of imaging is more than 5 cm, we believe that our study presents a compelling reason to use our system in real clinical situations. In the near future, we plan to conduct a clinical study in humans.

## Supplementary information

**Additional file 1: Figure S1.** Modeling of rats with orthotopic prostate cancer. A. Ultrasound images. B. General observation. Blue arrow: bladder. Yellow arrow: tumor. C. Observation under the microscope. Black arrow: prostate gland. Red arrow: cancerous tissue. (Magnitude: 50 ×). **Figure S2.** Pathology including both *H&E* staining and immunochemistry staining of both prostate gland and tumor tissue. (Magnitude: 100 ×). **Figure S3.** Representative PA map images of rats with orthotopic PCa.

## Data Availability

The datasets used and/or analysed during the current study available from the corresponding author on reasonable request.

## Abbreviations

PCa: Prostate cancer
US: Ultrasound
TRUS: Transrectal ultrasound
MpMRI: Multiparametric Magnetic Resonance Imaging
PAI: Photoacoustic imaging
RGD: Arginine-glycine-aspartic acid sequence
cRGD: Cyclic arginine-glycine-aspartic acid sequence
DMSO: Dimethyl sulfoxide
UV-Vis: Ultraviolet-visible
HUVEC: Human umbilical vein endothelial cells
293 T: Human embryonic kidney cells
FBS: Fetal bovine serum
