# Supplementary material for: Targeted imaging of orthotopic prostate cancer by using clinical transformable photoacoustic molecular probe
Source: BMC Cancer. 2020 May 14;20:419. doi: 10.1186/s12885-020-06801-9 (PMC7222516; doi:10.1186/s12885-020-06801-9)

**Supplemental Materials and Methods**

***Histopathological Analysis of Rat Prostate Cancer***

After imaging, prostate tissues were excised, formalin-fixed and paraffin-embedded. Tissue sections (10 μm) were stained with Hematoxylin and Eosin (*H&E*) following standard protocol. Before *H&E* staining, samples were fixed with freshly prepared 10% PBS buffered formalin for 24 h. After deparaffinization, 3 μm of prostate tissue section was cut for *H&E* staining. Cancer was defined as extensive proliferation into a solid mass with little or no normal gland. The *α_v_β_3_* integrin expression was determined by immunohistochemical staining via the standard procedure. After deparaffinization with xylene and alcohol, prostate tissues were incubated in 10 mM citrate buffer (pH 7.4) for 10 minutes at 90 °C for antigen retrieval. The 0.3% H_2_O_2_ in methanol was added to the samples at 4 °C for 30 minutes to inactivate the endogenous peroxidases. After incubation with 0.5% Triton for 10 minutes, the sections are treated with 10% normal goat serum for 1 h at room temperature for antigen blocking. Polyclonal antibody (Abcam, England) was then added to the sections for 1 h at 37 °C. Finally, after being washed with PBS, all sections were incubated with horseradish conjugated biotinylated secondary anti-mouse antibody (Vector Laboratories, America) for 1 h. The immuno-reactivity on the tissue sections was visualized using the peroxidase substrate DAB. The nuclei were counterstained by hematoxylin.

***CEUS imaging of Prostate Cancer***

Three days after the operation of orthotopic PCa, CEUS was performed to image local PCa processing. Rats were first appropriately anesthetized. CEUS imaging was performed on a clinical US imaging system (Logiq E9 digital premium ultrasound system, GE, USA). Images were collected using a broadband ML6-15D high-frequency probe.

**Supplemental figures and tables**

**Figure S1**


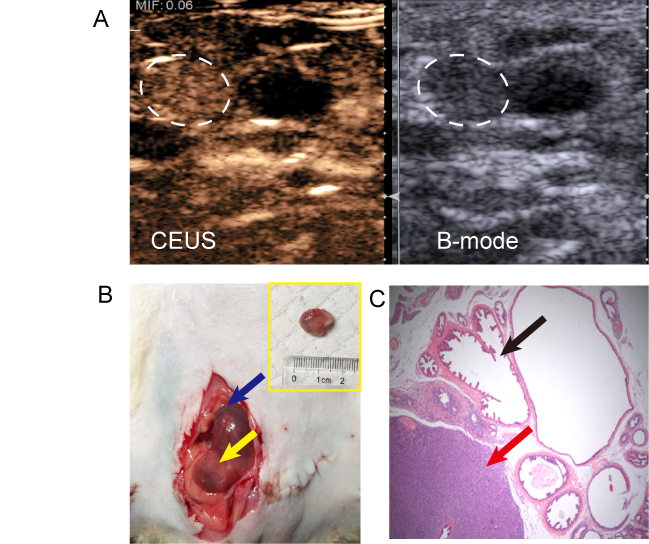


**Figure S2**


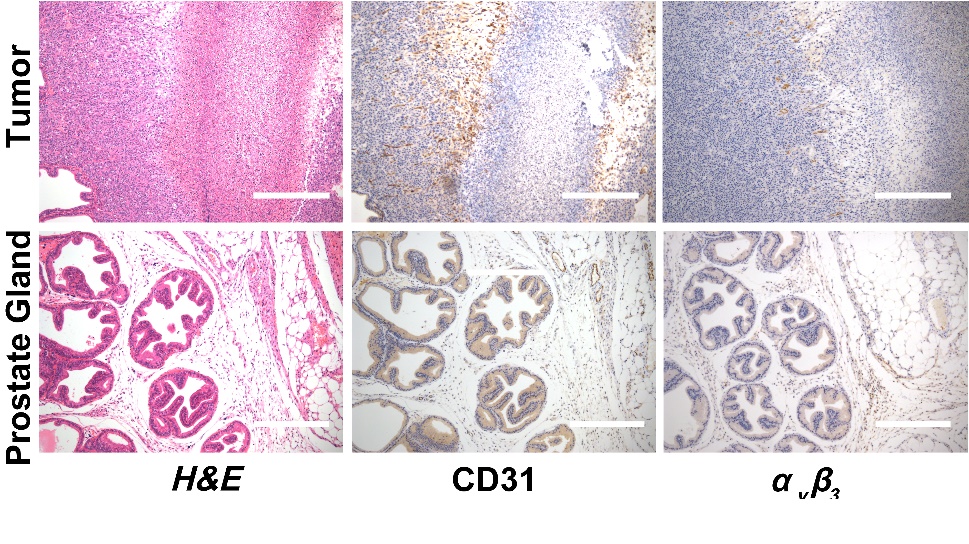


**Figure S3**


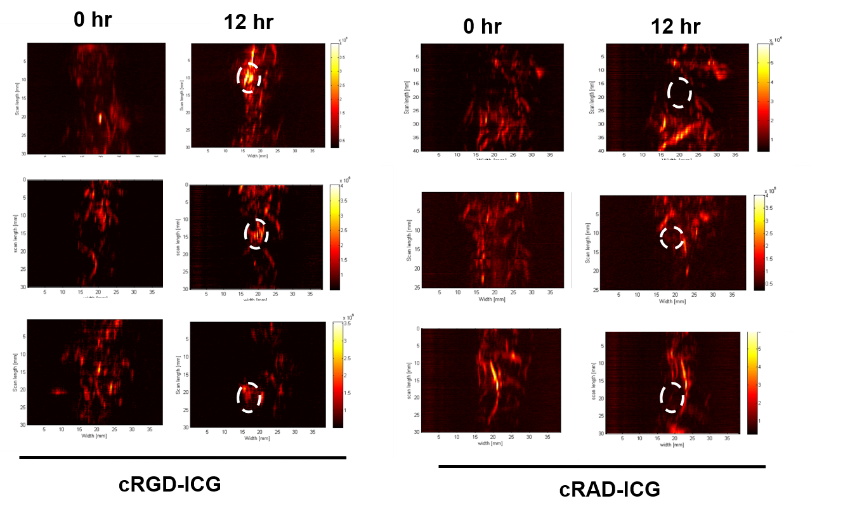

Supplement: Supplementary file 1 — Additional file 1: Figure S1. Modeling of rats with orthotopic prostate cancer. A. Ultrasound images. B. General observation. Blue arrow: bladder. Yellow arrow: tumor. C. Observation under the microscope. Black arrow: prostate gland. Red arrow: cancerous tissue. (Magnitude: 50 ×). Figure S2. Pathology including both H&E staining and immunochemistry staining of both prostate gland and tumor tissue. (Magnitude: 100 ×). Figure S3. Representative PA map images of rats with orthotopic PCa. [file 12885_2020_6801_MOESM1_ESM.docx]
